# ZFYVE1 negatively regulates MDA5- but not RIG-I-mediated innate antiviral response

**DOI:** 10.1371/journal.ppat.1008457

**Published:** 2020-04-06

**Authors:** Xuan Zhong, Lu Feng, Ru Zang, Cao-Qi Lei, Qing Yang, Hong-Bing Shu

**Affiliations:** Department of Infectious Diseases, Zhongnan Hospital of Wuhan University, Frontier Science Center for Immunology and Metabolism, Medical Research Institute, Wuhan University, Wuhan, China; University of Pennsylvania, UNITED STATES

## Abstract

The retinoic acid-inducible gene-I (RIG-I)-like receptors (RLRs), including RIG-I and melanoma differentiation-associated gene 5 (MDA5), sense cytoplasmic viral RNA and initiate innate antiviral responses. How RIG-I and MDA5 are differentially regulated remains enigmatic. In this study, we identified the guanylate-binding protein (GBP) and zinc-finger FYVE domain-containing protein ZFYVE1 as a negative regulator of MDA5- but not RIG-I-mediated innate antiviral responses. ZFYVE1-deficiency promoted MDA5- but not RIG-I-mediated transcription of downstream antiviral genes. Comparing to wild-type mice, *Zfyve1*^*-/-*^ mice were significantly protected from lethality induced by encephalomyocarditis virus (EMCV) that is sensed by MDA5, whereas *Zfyve1*^*-/-*^ and *Zfyve1*^*+/+*^ mice were comparable to death induced by vesicular stomatitis virus (VSV) that is sensed by RIG-I. Mechanistically, ZFYVE1 interacted with MDA5 but not RIG-I. ZFYVE1 bound to viral RNA and decreased the ligand binding and oligomerization of MDA5. These findings suggest that ZFYVE1 acts as a specific negative regulator of MDA5-mediated innate immune responses by inhibiting its ligand binding and oligomerization.

## Author summary

RIG-I and MDA5 are the main cytosolic sensors for invaded viral RNA. How these sensors are differentially regulated is largely unknown. In this study, we identified ZFYVE1 as a specific regulator of MDA5- but not RIG-I-mediated antiviral responses. ZFYVE1-deficiency promotes antiviral immune responses and renders the mice less susceptible to EMCV-induced death. ZFYVE1 interacts with MDA5 and viral dsRNA, and inhibits the ligand binding and oligomerization of MDA5. Our study reveals a negative regulatory mechanism for keeping MDA5 inactive in un-infected cells, which contributes to our understanding on how innate antiviral responses are delicately regulated to avoid immune damage.

## Introduction

The innate immune system is the first line of host defense against microbial infection. Upon viral infection, the structurally conserved viral molecules called pathogen-associated molecular patterns (PAMPs) are sensed by pathogen recognition receptors (PRRs). The PRRs then trigger a series of signaling events, leading to the induction of type I interferons (IFNs), proinflammatory cytokines and other downstream antiviral effector proteins. These effectors act to inhibit viral replication, clear the infected cells and facilitate adaptive immune responses [1].

During viral infection, viral nucleic acids act as important PAMPs. It has been demonstrated that the membrane-associated Toll-like receptor 3 (TLR3) recognizes extracellular and endosomal viral RNA, whereas the cytosolic viral RNA is detected by retinoic acid-inducible gene-I (RIG-I) like receptors (RLRs), including RIG-I and melanoma differentiation-associated gene 5 (MDA5) [2]. Upon binding to their ligands, RIG-I and MDA5 undergo conformational changes and recruit PP1α/γ for their dephosphorylation, which is followed by their K63-linked polyubiquitination by several E3 ubiquitin ligases [3–7]. The RLRs then form longer filaments on viral RNA and then interact with the mitochondrial adaptor protein VISA (also called MAVS, IPS-1, and Cardif) through their respective N-terminal caspase activation and recruitment domains (CARDs) [7–12]. VISA acts as a central platform for assembly of a virus-induced complex that activates TAK1-IKK and TBK1/IKKε kinases, leading to activation of the transcription factors NF-κB and IRF3 and ultimate induction of downstream antiviral genes [11].

Although RIG-I and MDA5 share certain sequence, structural and functional similarities, they have distinct structural and functional properties and are regulated by distinct post-translational modifications [13]. For example, RIG-I but not MDA5 exhibits an auto-inhibition state through the intramolecular interaction of its CARDs and the C-terminal tail domain (CTD) in un-infected cells [14]. RIG-I recognizes 5’-triphosphorylated (PPP) blunt-ended double-stranded (ds) RNA or single-stranded (ss) RNA hairpins present in a variety of positive and negative strand viruses such as influenza virus, Sendai virus (SeV) and vesicular stomatitis virus (VSV) [15–17]. MDA5 primarily recognizes relatively long dsRNA in the genomes of dsRNA viruses or dsRNA replication intermediates of positive-strand viruses, such as encephalomyocarditis virus (EMCV) and poliovirus [18]. The genomes of some RNA viruses, such as West Nile virus and dengue virus, can be recognized by both RIG-I and MDA5 [19]. Both RIG-I and MDA5 can recognize the synthetic viral dsRNA analog poly(I:C), but with length dependency. The high molecular weight (HMW) poly(I:C) (>3 kb) is recognized by MDA5, whereas the low molecular weight (LMW) poly(I:C) (<1.5 kb), ss or ds RNA with 5’-PPP group is primarily recognized by RIG-I [20].

Although it has been well established that RIG-I and MDA5 sense RNAs originated from different types of viruses. Whether and how these sensors are distinctly regulated is largely unknown. In this study, we identified the guanylate-binding protein (GBP) and zinc-finger FYVE domain-containing protein ZFYVE1 as a negative regulator of MDA5- but not RIG-I-mediated antiviral responses. ZFYVE1 specifically regulates MDA5-mediated innate immune responses by impairing its viral RNA binding and oligomerization. Our findings reveal a mechanism for distinct regulation of the RLR members and provide insight to delicate regulations of innate antiviral responses to avoid autoinflammatory responses.

## Results

### ZFYVE1-deficiency enhances MDA5-mediated signaling

**Fig 1:**
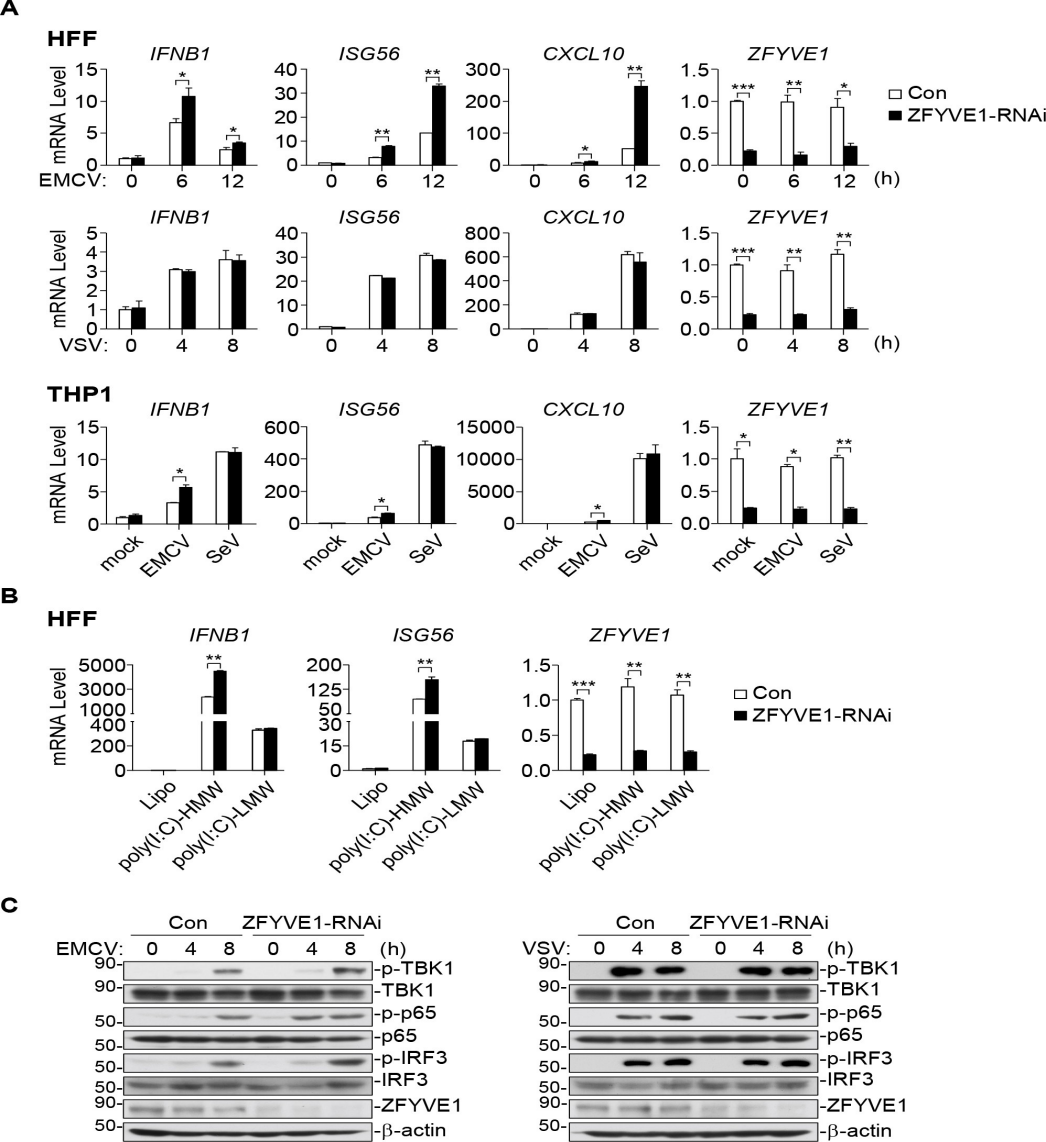
Identification of ZFYVE1 as a negative regulator of MDA5-triggered signaling. (A) Knockdown of ZFYVE1 enhances transcription of downstream antiviral genes induced by EMCV but not SeV or VSV. HFFs or THP1 cells (1 × 10^5^) stably transduced with control or ZFYVE1-RNAi plasmids were left un-infected or infected with EMCV, VSV or SeV for the indicated times (HFFs) or 6 h (THP1) before qPCR analysis of mRNA levels of the indicated genes. (B) Knockdown of ZFYVE1 enhances transcription of downstream antiviral genes induced by transfection of poly(I:C)-HMW but not poly(I:C)-LMW. HFFs (1 × 10^5^) stably transduced with control or ZFYVE1-RNAi plasmids were mock- transfected or transfected with poly(I:C)-HMW (2 μg/ml) or poly(I:C)-LMW (2 μg/ml) for 4 h before qPCR analysis of mRNA levels of the indicated genes. (C) Knockdown of ZFYVE1 enhances EMCV- but not VSV-induced phosphorylation of TBK1, IRF3, and p65. HFFs (1 × 10^5^) stably transduced with control or ZFYVE1-RNAi were left un-infected or infected with EMCV or VSV for the indicated times before immunoblotting analysis with the indicated antibodies. *P < 0.05, **P < 0.01 and ***P < 0.001 (unpaired t test). Data shown are mean ± SD, n = 3 (technical replicate, A and B), and representative of three biological replicates (A-C) with similar results.

Previously, we have reported that ZFYVE1 modulates TLR3-mediated signaling by facilitating its ligand binding [21]. We further investigated the functions of ZFYVE1 in RLR-mediated signaling. Quantitative PCR (qPCR) analysis indicated that knockdown of ZFYVE1 potentiated EMCV- but not SeV- or VSV-triggered transcription of *IFNB1*, *ISG56* and *CXCL10* genes in human foreskin fibroblasts (HFFs) and THP1 cells (Fig 1A). Knockdown of ZFYVE1 enhanced the transcription of *IFNB1*, *ISG56* genes induced by transfected cytoplasmic poly(I:C)-HMW but not poly(I:C)-LMW (Fig 1B). In addition, knockdown of ZFYVE1 enhanced EMCV- but not VSV-triggered phosphorylation of TBK1, IRF3 and p65, which are hall-marks of IRF3 and NF-κB activation (Fig 1C). As EMCV RNA and poly(I:C)-HMW are specifically sensed by MDA5, while VSV RNA, SeV RNA and poly(I:C)-LMW are mostly sensed by RIG-I [18, 19]. These results suggest that ZFYVE1 is a negative regulator of MDA5- but not RIG-I-mediated signaling.

**Fig 2:**
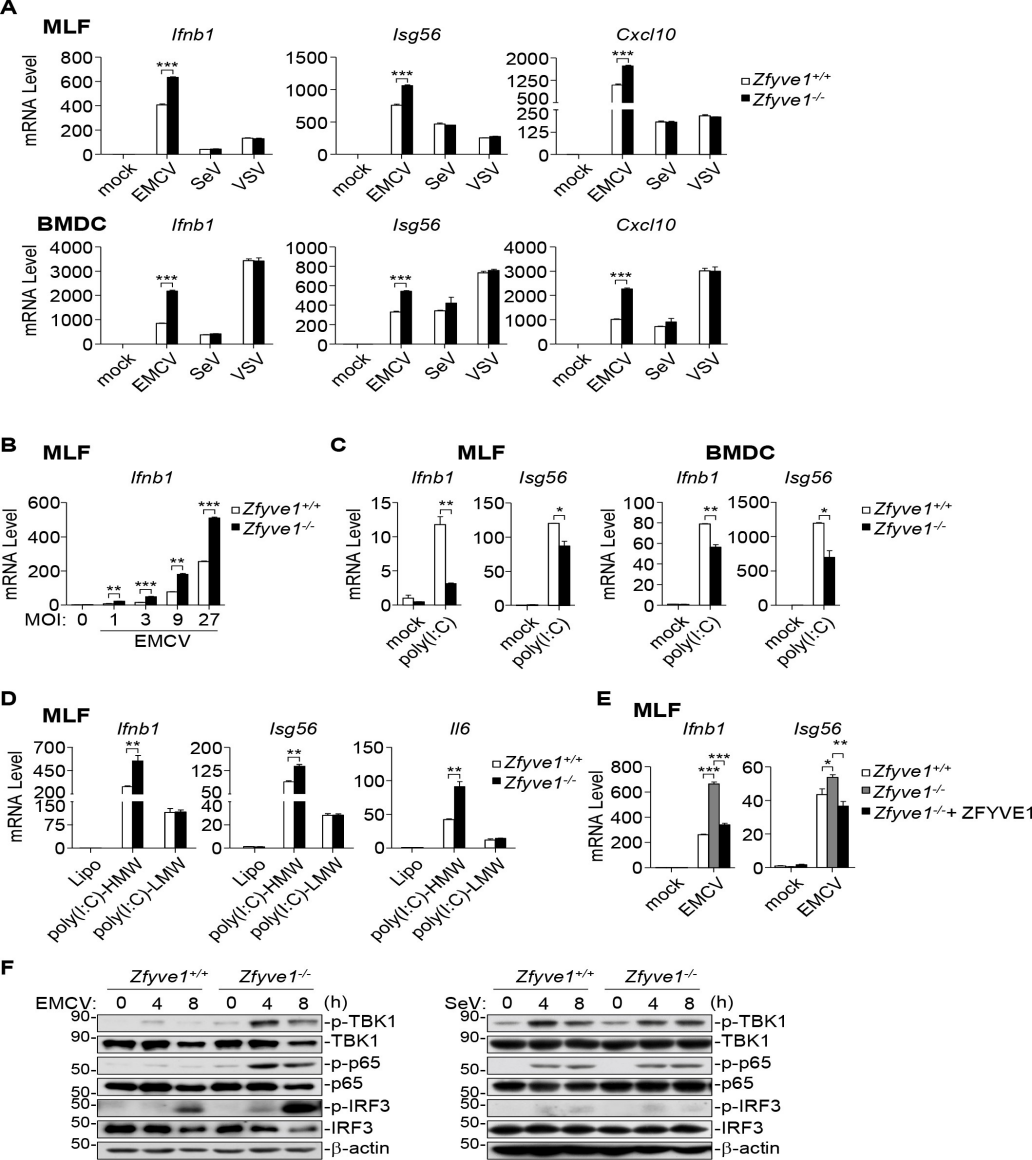
Zfyve1-deficiency enhances MDA5-mediated signaling in primary mouse cells. (A) Zfyve1-deficiency enhances transcription of downstream antiviral genes induced by EMCV but not SeV or VSV in mouse cells. *Zfyve1*^*+/+*^ and *Zfyve1*^*-/-*^ MLFs or BMDCs (2 × 10^5^) were left un-infected or infected with EMCV, SeV or VSV for 6 h before qPCR analysis. (B) Zfyve1-deficiency increases transcription of *Ifnb1* gene induced by various doses of EMCV. *Zfyve1*^*+/+*^ and *Zfyve1*^*-/-*^ MLFs (2 × 10^5^) were left un-infected or infected with EMCV at MOI of 1, 3, 9 and 27 for 6 h before qPCR analysis. (C) Zfyve1-deficiency inhibits TLR3-mediated transcription of downstream genes. *Zfyve1*^*+/+*^ and *Zfyve1*^*-/-*^ MLFs (2 × 10^5^) were left un-treated or treated with poly(I:C) (50 μg/ml) in the culture medium for 2 h before qPCR analysis. (D) Zfyve1-deficiency enhances transcription of downstream antiviral genes induced by transfection of poly(I:C)-HMW but not poly(I:C)-LMW in MLFs. *Zfyve1*^*+/+*^ and *Zfyve1*^*-/-*^ MLFs (2 × 10^5^) were transfected with lipo, poly(I:C)-LMW (2 μg/ml) or poly(I:C)-HMW (2 μg/ml) for 4 h before qPCR analysis. (E) Reconstitution of ZFYVE1 in *Zfyve1*^*-/-*^ inhibits EMCV-induced transcription of downstream antiviral genes. *Zfyve1*^*-/-*^ MLFs were reconstituted with ZFYVE1 by lentiviral-mediated gene transfer. The reconstituted MLFs were left un-infected or infected with EMCV for 6 h before qPCR analysis. (F) Zfyve1-deficiency enhances EMCV- but not SeV-induced phosphorylation of TBK1, IRF3, and p65. *Zfyve1*^*+/+*^ and *Zfyve1*^*-/-*^ MLFs (2 × 10^5^) were left un-infected or infected with EMCV or SeV for the indicated times before immunoblotting analysis with the indicated antibodies. *P < 0.05, **P < 0.01 and ***P < 0.001 (unpaired t test). Data shown are mean ± SD, n = 3 (technical replicate, A-E), and representative of three biological replicates (A-F) with similar results.

To investigate the functions of ZFYVE1 *in vivo*, we generated ZFYVE1-deficient mice by CRISPR-Cas9 technology [22, 23]. We prepared mouse lung fibroblasts (MLFs) and bone marrow-derived dendritic cells (BMDCs) from *Zfyve1*^*+/+*^ and *Zfyve1*^*-/-*^ mice. qPCR analysis indicated that ZFYVE1-deficiency enhanced EMCV- but not SeV- or VSV-induced transcription of *Ifnb1*, *Isg56*, and *Cxcl10* genes in MLFs and BMDCs (Fig 2A). The folds of increase of virus-induced transcription of *Ifnb1* gene in *Zfyve1*^*-/-*^ MLFs were comparable when the cells were infected with different doses of EMCV (Fig 2B). Consistent with our previous study [21], transcription of downstream genes including *Ifnb1* and *Isg56* induced by stimulation of TLR3 with poly(I:C) in the medium was inhibited in Zfyve1-deficient cells in comparison to their wild-type counterparts (Fig 2C). Consistently, the transcription of *Ifnb1*, *Isg56* and *Il6* genes induced by transfected cytoplasmic poly(I:C)-HMW but not poly(I:C)-LMW was enhanced in *Zfyve1*^*-/-*^ MLFs in comparison with their wild-type counterparts (Fig 2D). Reconstitution of ZFYVE1 in *Zfyve1*^-/-^ MLFs inhibited EMCV-induced transcription of *Ifnb1*, *Isg56* genes (Fig 2E). In addition, ZFYVE1-deficiency also enhanced EMCV- but not SeV-induced phosphorylation of TBK1, IRF3 and p65 in MLFs (Fig 2F). These data suggest that ZFYVE1-deficiency specifically promotes MDA5- but not RIG-I-mediated signaling in primary mouse cells.

### ZFYVE1-deficiency enhances MDA5-mediated innate antiviral response *in vivo*

**Fig 3:**
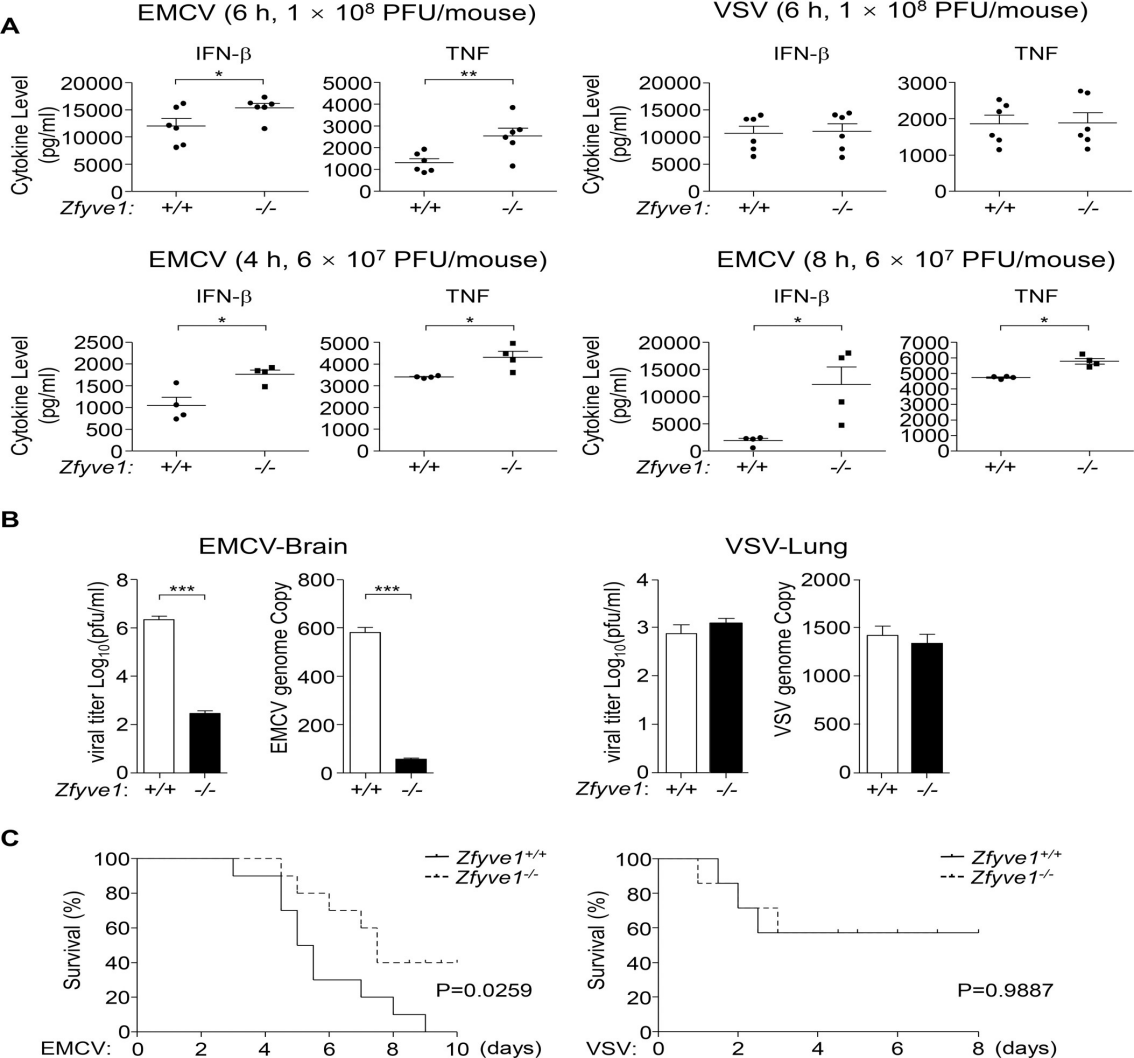
ZFYVE1-deficiency enhances MDA5-mediated innate antiviral response *in vivo*. (A) Zfyve1-deficiency increases EMCV- but not VSV-induced serum levels of IFN-β and TNF. Sex- and age-matched *Zfyve1*^*+/+*^ and *Zfyve1*^*-/-*^ mice (n = 4–6 for each viral dose/time point/genotype group) were infected intraperitoneally (i.p.) with the indicated doses of EMCV or VSV for the indicated times before serum cytokines were measured by ELISA. (B) Zfyve1-deficiency inhibits viral replication in the brains of EMCV-infected mice. Sex- and age-matched *Zfyve1*^*+/+*^ and *Zfyve1*^*-/-*^ mice (n = 3 for each genotype group) were infected intraperitoneally (i.p.) with EMCV (1 × 10^8^ PFU) or VSV (1 × 10^8^ PFU) for 2 days. Viral titers and genomic copy numbers in the brains of EMCV-infected mice or the lungs of VSV-infected mice were quantified by plaque assays and qPCR respectively. (C) Zfyve1-deficient mice are less susceptible to EMCV-induced death. Sex- and age-matched *Zfyve1*^*+/+*^ and *Zfyve1*^*-/-*^ mice were infected intraperitoneally (i.p.) with EMCV (6 × 10^7^ PFU per mouse, n = 10 for each genotype group) or VSV (1 × 10^8^ PFU per mouse, n = 6 for each genotype group), and mouse survival was observed and recorded for 10 or 8 days. *P < 0.05, **P < 0.01 and ***P < 0.001 (unpaired t test in A-B or log-rank test in C). Data shown are mean ± SEM.

To further explore the roles of ZFYVE1 in regulation of MDA5-mediated innate immune responses *in vivo*, age- and sex-matched *Zfyve1*^*+/+*^ and *Zfyve1*^*-/-*^ mice were infected with EMCV and VSV through intraperitoneal (i.p.) route. As shown in Fig 3A, sera from *Zfyve1*^*-/-*^ mice infected with different doses of EMCV showed higher levels of IFN-β and TNF compared with that of wild-type mice at all examined time points, whereas VSV-triggered serum cytokine levels were comparable between *Zfyve1*^*+/+*^ and *Zfyve1*^*-/-*^ mice. Consistently, EMCV genomic copies and viral titers in the brain of *Zfyve1*^*-/-*^ mice after infection were significantly decreased compared to those of *Zfyve1*^*+/+*^ mice (Fig 3B). In similar experiments, VSV genomic copies and viral titers in the lungs of *Zfyve1*^*-/-*^ mice after infection were comparable to those of *Zfyve1*^*+/+*^ mice (Fig 3B). In addition, we found that *Zfyve1*^*-/-*^ mice were more resistant to EMCV-induced death compared to the wild-type mice, whereas they were comparable to VSV-induced death (Fig 3C). These results suggest that ZFYVE1 inhibits MDA5- but not RIG-I-mediated innate immune responses in mice.

### ZFYVE1 is associated with MDA5

**Fig 4:**
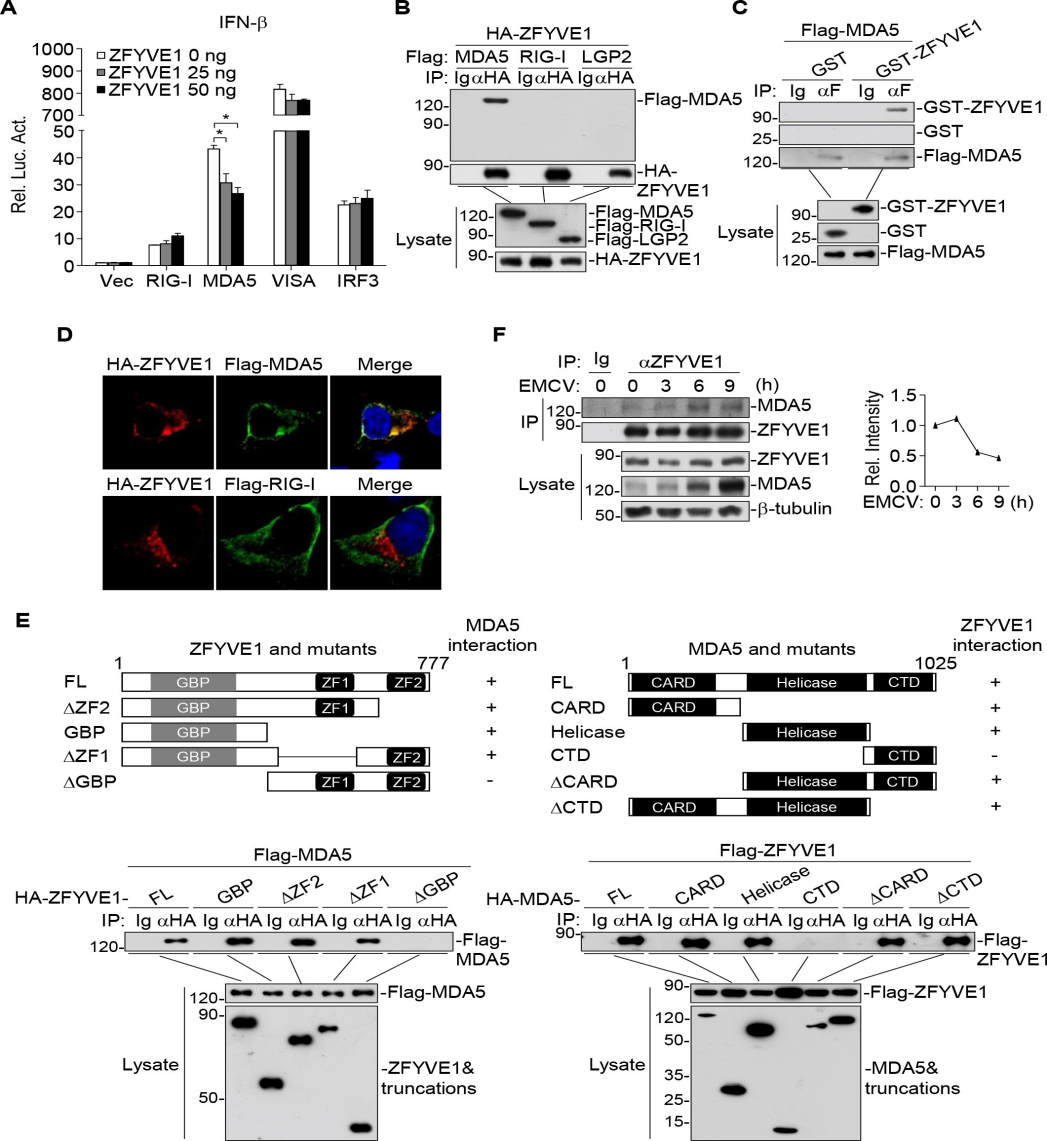
ZFYVE1 is associated with MDA5. (A) Overexpression of ZFYVE1 inhibits activation of the IFN-β promoter mediated by MDA5 but not RIG-I, VISA or IRF3. HEK293 cells were transfected with the IFN-β promoter reporter (0.05 μg), the indicated expression plasmids (0.1 μg), and increased amounts of ZFYVE1 plasmid (0.025, 0.05 μg) for 24 h before luciferase assays. (B) ZFYVE1 is associated with MDA5 but not RIG-I and LGP2. HEK293 cells (2 × 10^6^) were transfected with the indicated plasmids (5 μg each) for 20 h before co-immunoprecipitation and immunoblotting analysis. (C) ZFYVE1 interacts with MDA5 *in vitro*. HEK293 cells were transfected with Flag-MDA5 for 24 h. The lysates were incubated with anti-Flag agarose beads for 2 h at 4°C. The beads were washed three times with lysis buffer and then Flag-MDA5 was eluted with 3 × Flag peptides in 250 mM Tris-HCl, pH 8.0. The purified Flag-MDA5 was mixed with bacterially-expressed GST-ZFYVE1 or GST (5 μg each). The mixtures were further immunoprecipitated with anti-Flag for 2 h, followed by immunoblotting analysis with the indicated antibodies. (D) ZFYVE1 is co-localized with MDA5 but not RIG-I. HEK293 cells (1 × 10^5^) were transfected with expression plasmids for HA-ZFYVE1 (0.5 μg) and Flag-MDA5 (1 μg) or Flag-RIG-I (1 μg). Twenty hours later, the cells were fixed with 4% paraformaldehyde for 10 min, and permeabilized with 0.1% Triton X-100 in PBS for 15 min. The cells were blocked with 1% BSA in PBS and stained with the primary (anti-ZFYVE1 and anti-Flag) and secondary antibodies. the nuclei were stained with DAPI. The cells were observed with a Zeiss confocal microscope with a 60 × objective. (E) Domain mapping of the interaction between ZFYVE1 and MDA5. HEK293 cells (2 × 10^6^) were transfected with the indicated plasmids for 20 h before co-immunoprecipitation and immunoblotting analysis. (F) Endogenous ZFYVE1 is associated with MDA5. THP1 cells (4 × 10^7^) were left un-infected or infected with EMCV for the indicated times before co-immunoprecipitation and immunoblotting analysis. The right graph shows the relative amounts of ZFYVE1-associated MDA5, which were quantitated using ImageJ and normalized to the total MDA5 levels in the lysates. *p < 0.05 (unpaired t test). Data shown are mean ± SD, n = 3 (technical replicate, A), and representative of three biological replicates (A-F) with similar results.

We next investigated the molecular mechanisms responsible for the inhibitory effects of ZFYVE1 on MDA5-mediated innate immune responses. Reporter assays indicated that overexpression of ZFYVE1 significantly inhibited activation of the IFN-β promoter mediated by overexpression of MDA5 but not RIG-I, VISA or IRF3 (Fig 4A). Transient transfection and co-immunoprecipitation experiments indicated that ZFYVE1 was associated with MDA5 but not RIG-I and LGP2 (Fig 4B). Co-immunoprecipitation experiments with purified recombinant proteins indicated that ZFYVE1 directly interacted with MDA5 (Fig 4C). Confocal microscopy indicated that ZFYVE1 was co-localized with MDA5 but not RIG-I (Fig 4D). Domain mapping experiments indicated that the N-terminal GBP but not the C-terminal FYVE domain of ZFYVE1 was responsible for its interaction with MDA5. On the other hand, the N-terminal tandem CARDs and the middle helicase domain of MDA5 could independently interact with ZFYVE1 (Fig 4E). Endogenous co-immunoprecipitation experiments indicated that ZFYVE1 was constitutively associated with MDA5 in un-infected cells (Fig 4F). Although the association of ZFYVE1 with MDA5 was slightly increased following EMCV infection because of the dramatic induction of MDA5, their affinity was decreased and more MDA5 was not associated with ZFYVE1 after viral infection (Fig 4F). These results suggest that ZFYVE1 targets MDA5 to exert its functions.

### ZFYVE1 competes with MDA5 for viral RNA binding

**Fig 5:**
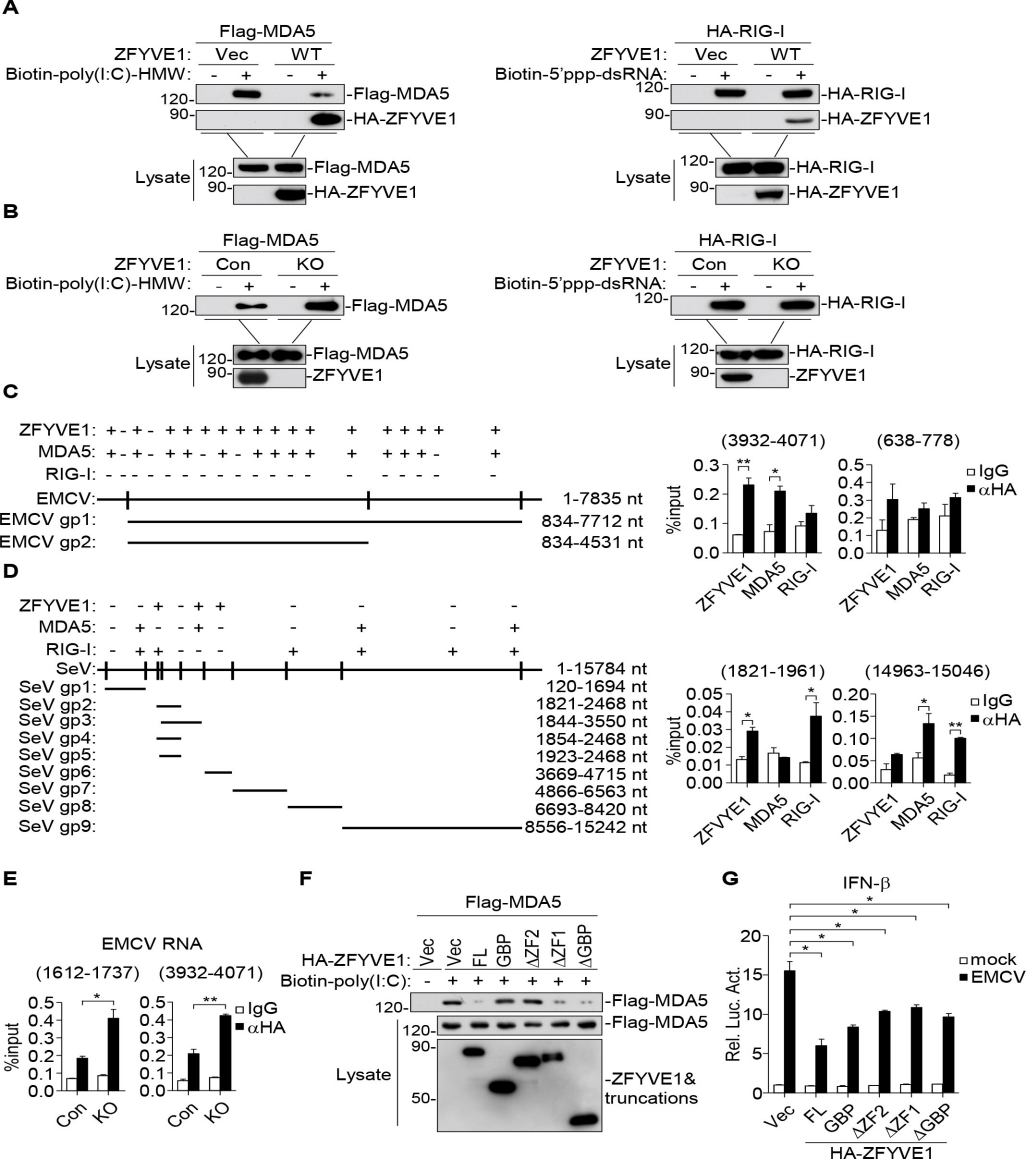
ZFYVE1 competes with MDA5 for viral RNA binding. (A) ZFYVE1 inhibits the binding of MDA5 to poly(I:C)-HMW. HEK293 cells (2 × 10^6^) were transfected with the indicated plasmids (5 μg each). The lysates were incubated with biotinylated-poly(I:C)-HMW or biotinylated-5'ppp-dsRNA at 37°C for 1 h, and then incubated with streptavidin agarose at 37°C for another 2 h. The RNA-binding proteins were analyzed by immunoblots with anti-Flag and anti-HA. (B) ZFYVE1-deficiency enhances the binding of MDA5 to poly(I:C)-HMW. ZFYVE1-KO and control HEK293 cells (2 × 10^6^) were transfected with an expression plasmid for Flag-MDA5 or HA-RIG-I (5 μg each). Twenty hours after transfection, cells were collected for pull-down assays similarly as in (A). (C) ZFYVE1 and MDA5 but not RIG-I bind to EMCV RNA. HEK293 cells were transfected with the indicated HA-tagged plasmids (5 μg each) for 20 h, then infected with EMCV for 1 h, washed with medium and cultured at 37°C for additional 2 h. Cell lysates were immunoprecipitated with IgG or anti-HA (5 μg) and protein G beads (50 μl) at 4°C for 2 h. The immunoprecipitates were treated with diluted RNase I (1:25 in PBS) at 37°C for 5 min. The bead-bound immunoprecipitates were washed for three times with lysis buffer containing RNase inhibitors. The protein and RNA complexes were eluted with 200 μl TE buffer containing 10 mM DTT at 37°C for 30 min. The protein-bound RNAs were extracted and analyzed by qPCR analysis with primers corresponding to the indicated regions of EMCV genome. Positive (+) and negative (-) detections were shown at the top of the schematic presentation of the EMCV genome. (D) ZFYVE1, MDA5 and RIG-I bind to SeV RNA. HEK293 cells (2 × 10^6^) were transfected with the indicated plasmids (5 μg each) for 20 h, and then the cells were infected with SeV for 1 h. Cell lysates were collected for “footprint” experiments similarly as in (C). Positive (+) and negative (-) detections were shown at the top of the schematic presentation of the SeV genome. (E) ZFYVE1-deficiency enhances the binding of MDA5 to EMCV RNA. ZFYVE1-KO and control HEK293 cells (2 × 10^6^) were transfected with the indicated plasmids (5 μg each). At 20 h after transfection, cells were infected with EMCV for 1 h. Cell lysates were collected for “footprint” experiments similarly as in (C). (F) The C-terminal FYVE domain of ZFYVE1 is important for inhibition of MDA5 binding to poly(I:C)-HMW. HEK293 cells (2 × 10^6^) were transfected with the indicated plasmids (5 μg each). Eighteen hours later, cells were collected for *in vitro* pull-down assays similarly as in (A). (G) Overexpression of ZFYVE1 and its mutants inhibit EMCV-triggered activation of the IFN-β promoter. HT1080 cells were transfected with the IFN-β promoter reporter (0.2 μg) and ZFYVE1 or its mutant plasmids (0.2 μg) for 24 h, and then left un-infected or infected with EMCV for 12 h before luciferase assays. *P < 0.05 and **P < 0.01 (unpaired t test). Data shown are mean ± SD, n = 3 (technical replicate, C-E and G) and representative of three biological replicates (A-G) with similar results.

ZFYVE1 has been reported as a poly(I:C) binding protein in our previous study [21]. We wondered whether ZFYVE1 affects the binding of MDA5 to poly(I:C). Pull-down experiments showed that overexpression of ZFYVE1 inhibited the binding of MDA5 to poly(I:C)-HMW, but had no marked effects on the binding of RIG-I to 5’ppp-dsRNA (Fig 5A). ZFYVE1-deficiency enhanced the binding of MDA5 to poly(I:C)-HMW but not the binding of RIG-I to poly(I:C)-LMW (Fig 5B). We next examined whether ZFYVE1 binds to viral RNA by “footprint” experiments [24, 25]. Following viral infection, we immunoprecipitated ZFYVE1, MDA5 or RIG-I, and the protein-bound viral RNA was detected by qPCR with primers targeting various regions of the viral genome. The results indicated that both ZFYVE1 and MDA5 but not RIG-I could bind to overlapping regions of EMCV RNA (Fig 5C & S1A Fig), while ZFYVE1, MDA5 and RIG-I could bind to different regions of SeV RNA (Fig 5D & S1B Fig). Consistently, ZFYVE1-deficiency increased the binding of MDA5 to EMCV RNA (Fig 5E). These results suggest that ZFYVE1 can bind to invaded EMCV RNA and ZFYVE1 competes with MDA5 for viral RNA binding.

Deletion analysis showed that ZFYVE1, ZFYVE1(ΔZF1) and ZFYVE1(ΔGBP) could inhibit the binding of MDA5 to poly(I:C)-HMW, while ZFYVE1-GBP and ZFYVE1(ΔZF2) did not affect MDA5 binding to poly(I:C)-HMW (Fig 5F). These results suggest that the second ZF domain of ZFYVE1 is required for its ability to inhibit the binding of MDA5 to poly(I:C)-HMW. Reporter assays indicated that wild-type ZFYVE1 markedly inhibited EMCV-induced activation of the IFN-β promoter. Interestingly, all the tested mutants, including the C-terminal ZF-containing mutants which inhibit the ligand binding of MDA5, and the N-terminal GBP-containing mutants which do not inhibit the ligand binding of MDA5, inhibited EMCV-induced activation of the IFN-β promoter to similar degrees (Fig 5G). These results suggest that ZFYVE1 regulates MDA5-mediated signaling through its competing for viral RNA binding and an additional mechanism that is dependent on its N-terminal domain.

### ZFYVE1 inhibits MDA5 oligomerization

**Fig 6:**
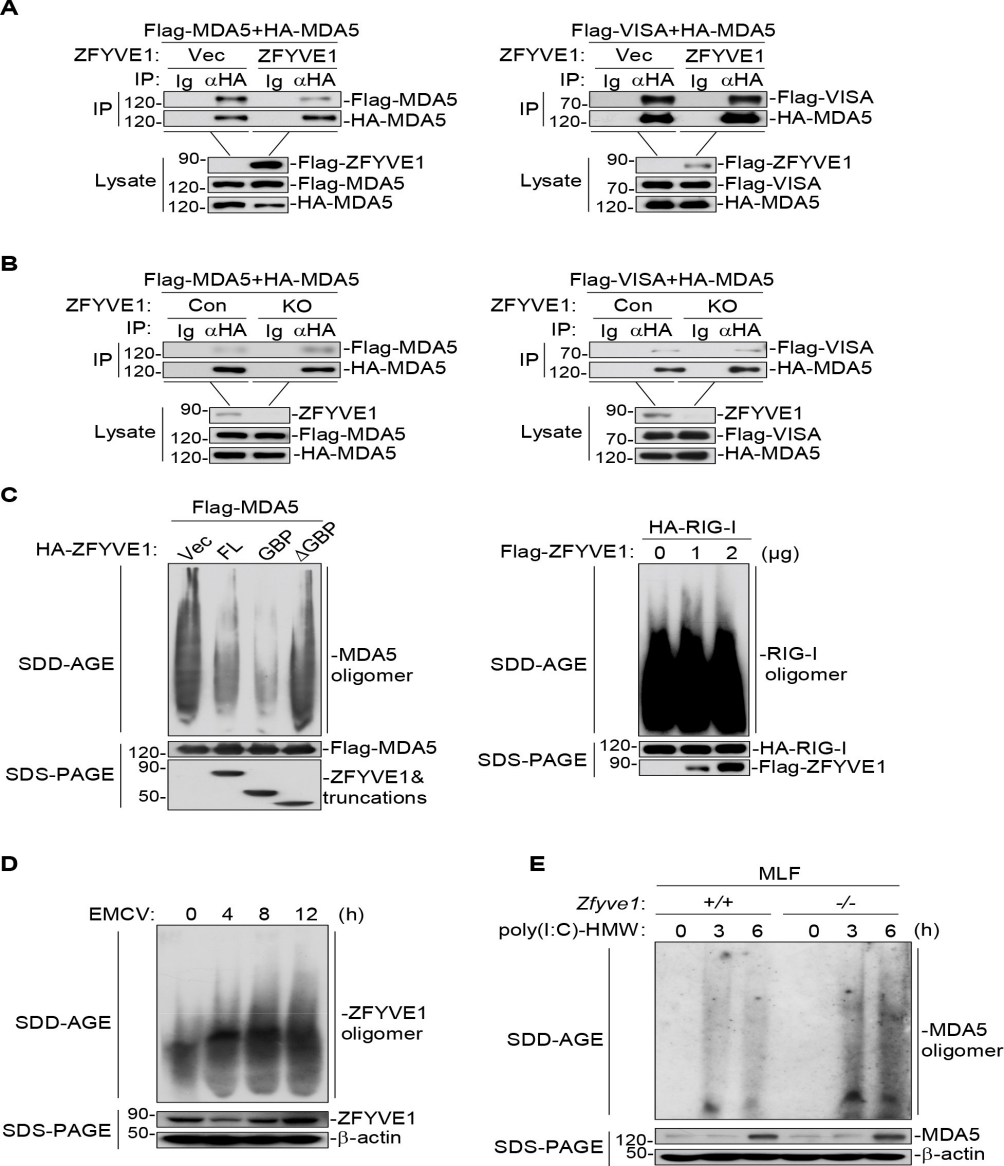
ZFYVE1 inhibits MDA5 oligomerization. (A) Overexpression of ZFYVE1 inhibits self-association of MDA5. HEK293 cells (2 × 10^6^) were transfected with the indicated plasmids (5 μg each) for 20 h before co-immunoprecipitation and immunoblotting analysis with the indicated antibodies. (B) ZFYVE1-deficiency enhances self-association of MDA5. ZFYVE1-KO and control HEK293 cells (2 × 10^6^) were transfected with the indicated plasmids (5 μg each) for 20 h before co-immunoprecipitation and immunoblotting analysis with the indicated antibodies. (C) The N-terminal GBP domain of ZFYVE1 is responsible for inhibition of MDA5 oligomerization. HEK293 cells (2 × 10^6^) were transfected with the indicated plasmids (10 μg each) for 20 h. Cell lysates were then fractionated by SDD-AGE and SDS-PAGE and analyzed by immunoblots with the indicated antibodies. (D) EMCV infection increases ZFYVE1 oligomerization. HT1080 cells (4 × 10^6^) were left un-infected or infected with EMCV for the indicated times. Cell lysates were then fractionated by SDD-AGE or SDS-PAGE and analyzed by immunoblots with the indicated antibodies. (E) ZFYVE1-deficiency enhances oligomerization of MDA5 induced by transfected poly(I:C)-HMW. *Zfyve1*^*+/+*^ and *Zfyve1*^*-/-*^ MLFs were transfected with poly(I:C)-HMW (4 μg/ml) for the indicated times. Cell lysates were then fractionated by SDD-AGE or SDS-PAGE and analyzed by immunoblots with the indicated antibodies. Data are representative of three biological replicates (A-E) with similar results.

It has been previously shown that the N-terminal tandem CARDs of MDA5 form functional oligomers which is important for VISA activation [12]. Co-immunoprecipitation experiments indicated that overexpression of ZFYVE1 markedly inhibited the self-association of MDA5, but had no marked effects on the association of MDA5 with VISA (Fig 6A). Conversely, ZFYVE1-deficiency enhanced self-association of MDA5, but had no marked effects on the association of MDA5 and VISA (Fig 6B). SDD-AGE experiments indicated that overexpression of ZFYVE1 inhibited the oligomerization of MDA5 but not RIG-I, which is a hallmark for their activation (Fig 6C). In these experiments, ZFYVE1-GBP but not ZFYVE1(ΔGBP) also inhibited MDA5 oligomerization (Fig 6C). In addition, EMCV infection increased the oligomerization of ZFYVE1 (Fig 6D), and the oligomerization of endogenous MDA5 induced by transfected poly(I:C)-HMW was increased in *Zfyve1*^*-/-*^ in comparison to *Zfyve1*^*+/+*^ MLFs (Fig 6E). These experiments suggest that ZFYVE1 inhibits MDA5 oligomerization through its N-terminal domain independent of its ability to inhibit the ligand binding of MDA5 through its C-terminal ZF domain.

## Discussion

The RLR family members RIG-I and MDA5 are key cytosolic sensors of viral RNAs, which play critical roles in innate immune responses to RNA viruses [26]. Although RIG-I and MDA5 share certain sequence, structural and functional similarities, they have distinct specificities to RNAs derived from different types of viruses. How these viral RNA sensors are differentially regulated is an important question to be answered. In the current study, we identified ZFYVE1 as a negative regulator of MDA5- but not RIG-I-mediated innate antiviral response.

Several lines of evidence suggest that ZFYVE1 specifically inhibits MDA5- but not RIG-I-mediated innate antiviral response. ZFYVE1-deficiency enhanced EMCV- and transfected poly(I:C)-HMW-induced transcription of downstream effector genes, but had no marked effects on SeV-, VSV- or transfected poly(I:C)-LMW-induced transcription of downstream effector genes in various human and murine cells. The serum cytokine levels induced by infection of EMCV were elevated in *Zfyve1*^-/-^ in comparison to wide-type mice, and ZFYVE1-deficient mice were less susceptible to EMCV- but not VSV-induced death. These results demonstrate that ZFYVE1 plays an important role in negative regulation of MDA5- but not RIG-I-mediated antiviral response. It would be interesting to further investigate the structural properties responsible for the specific inhibition of MDA5 but not RIG-I by ZFYVE1 in future studies.

Our results suggest that ZFYVE1 inhibits MDA5-mediated innate antiviral responses by competing with MDA5 for viral RNA binding and impairing MDA5 oligomerization. *In vitro pull*-down analysis as well as “footprint” experiments indicated that ZFYVE1 could bind to poly(I:C) and viral RNA in cells, which is dependent on its C-terminal ZF domain. Coimmunoprecipitation experiments indicated that ZFYVE1 was constitutively associated with MDA5 before and after viral infection. However, the affinity of ZFYVE1 with MDA5 was decreased and more MDA5 was not associated with ZFYVE1 following viral infection. In addition, viral infection induced oligomerization of ZFYVE1, while ZFYVE1 had an inhibitory role on virus-induced oligomerization of MDA5. Moreover, ZFYVE1 could compete with MDA5 for poly(I:C) or viral RNA binding, which inhibited EMCV-induced activation of IFN-β. The simplest explanation for our data is that upon viral infection ZFYVE1 competes with MDA5 for viral RNA binding. The binding of ZFYVE1 to viral RNA causes its oligomerization and conformational changes, which reliefs its inhibition of MDA5 (S2 Fig).

Recently, we have demonstrated that ZFYVE1 promotes TLR3-mediated innate immune response by promoting the ligand binding and activation of TLR3 [21]. It is possible that the distinct properties of TLR3 and MDA5, and/or the distinctly localized ZFYVE1 in the lumen of endo/lysosomes and cytosol are responsible for the distinct functions of ZFYVE1. Previously, it has been reported that DHX29 acts as a co-sensor of MDA5 for the recognition of viral dsRNA [27]. RAVER1 acts as a coactivator of MDA5-mediated cellular antiviral response by facilitating the binding of MDA5 to its ligand [28]. TRIM65 plays a critical role in MDA5-mediated signaling by promotes K63-linked polyubiquitination of MDA5 at lysine 743, which is critical for MDA5 oligomerization and activation [4]. 14-3-3η acts as a chaperone protein of MDA5 to promote antiviral innate immunity via facilitating MDA5 oligomerization and intracellular redistribution [29]. Our current study reveals a negative regulatory mechanism for keeping MDA5 inactive in un-infected cells, which contributes to our understanding on how innate antiviral response is delicately regulated to avoid immune damage.

## Materials and methods

### Ethics statement

The animal care and use were adhered to the Chinese National Guidelines for Ethical Review of Animal Welfare. The protocols and procedures for mice experiments in this study were approved by the Wuhan University College of Life Sciences Animal Care and Use Committee (approval number WDSKY0200902-2).

### Reagents, antibodies, cells and viruses

Trizol (Takara Bio), SYBR Green (Bio-Rad), RNase I (Ambion), Flag antibody-conjugated beads (Bimake), dual-specific luciferase assay kit (Promega), polybrene (Millipore), poly(I:C)-HMW (Invivogen), poly(I:C)-LMW (Invivogen), 5’ppp-dsRNA (Invivogen), DNAase I, 3 × Flag peptide (Sigma), Z-link Psoralen-PEG3-Biotin (Thermo), type II collagenase (Worthington), first-strand cDNA synthesis kit (Fermentas), GammaBind G Plus-Sepharose (Amersham Biosciences), and ELISA kits for murine IFN-β and TNF (BioLegend); mouse monoclonal antibodies for Flag and β-actin (Sigma), HA (Origene), p-IRF3, p65 and p-p65 (Cell Signaling Technology), IRF3 (Santa Cruz Biotechnology), p-TBK1 and TBK1 (Abcam), and ZFYVE1 (ABclonal); Alexa Fluor 488-conjugated goat anti-mouse IgG and Alexa Fluor 594-conjugated goat anti-rabbit IgG (Invitrogen); and HEK293 (American Type Culture Collection) and THP1 cells (American type culture collection) were purchased from the indicated companies. HFFs were provided by Dr. Min-Hua Luo (Wuhan Institute of Virology, CAS). SeV (Cantell strain), EMCV (BJC3 Strain) and VSV (Indiana Strain) were previously described [28, 30].

### Constructs

Genebank accession number for human ZFYVE1 is NM_021260. Mammalian expression plasmids for Flag-tagged LGP2, Flag-, HA- or GST-tagged ZFYVE1 and its mutants were constructed in the pRK5 or pMSCV vector [31] by standard molecular biology techniques. pRK vector-based mammalian expression plasmids for Flag- or HA-tagged MDA5 and its mutants, HA-RIG-I, Flag-VISA, and Flag-IRF3; and the IFN-β promoter reporter plasmid were previously described [25].

### ZFYVE1 knockout mice and genotyping

*Zfyve1* gene-knockout mice with a C57/B6J background were generated utilizing the CRISPR-Cas9 system by Wuhan University Animal Center. The exon 3 of ZFYVE1 was targeted by gRNAs, which resulted in a deletion in exon 3 (nt 484–988) of the mouse Zfyve1 coding sequence. Genotyping was performed by PCR using the following pairs of primers: 1F-TCTGGTGAGTAGACAGTGGTG, 1R-AGGCATAATCACTCTGCC TGG; 2F-AGCTGATTGCTGAAGTGAAAC, 2R-AGGCATAATCACTCTG CCTGG. Amplification of the WT allele with primer pair 1 resulted in a 250-bp fragment, whereas amplification of the disrupted allele with primer pair 2 resulted in a 450-bp fragment.

### Semi-denaturing detergent agarose gel electrophoresis (SDD-AGE)

SDD-AGE was performed as previously described [32, 33]. In brief, cell lysates were resuspended in 1 × sample buffer (0.5 × TBE, 10% glycerol, 2% SDS, and 0.0025% bromophenol blue) and loaded onto a vertical 1.5% agarose gel (Bio-Rad). After electrophoresis in running buffer (1 × TBE and 0.01% SDS) for 50 min with a constant voltage 100 V at 4°C, the proteins were transferred to immobilon membrane (Millipore) for immunoblotting analysis.

### Preparation of BMDCs

Bone marrow cells were isolated from tibiae and femur of mice. For preparation of BMDCs, bone marrow cells (1 × 10^7^) were cultured in RPMI 1640 medium supplemented with 10% FBS and murine GM-CSF (10 ng/ml) for 6–9 days.

### Preparation of MLFs

Primary MLFs were prepared from approximately 6- to 8-week-old mice as described [34]. The lungs were minced and digested in calcium and magnesium free HBSS containing 10 μg/ml type II collagenase and 20 μg/ml DNase I at 37°C for 3 h with shaking. Cell suspensions were filtered through progressively smaller cell strainers (100 and 40 μm) and then centrifuged at 1,500 rpm for 4 min. The cells were then plated in culture medium (1:1 [v/v] DMEM/Ham’s F-12 containing 10% FBS, 15 mM HEPES, 2 mM L-glutamine, 50 U/ml penicillin, and 50 μg/ml streptomycin). One hour later, adherent fibroblasts were rinsed with HBSS and cultured in Dulbecco's modified Eagle's medium (DMEM) supplemented with 10% FBS.

### Stable cell lines

HEK293 cells plated on 100-mm dishes were transfected with the indicated retroviral plasmids (10 μg) together with the pGag-pol (10 μg) and the pVSV-G (3 μg) plasmids. Two days after transfection, the viruses in the medium were harvested and used to infect THP1, MLFs and HFFs in the presence of polybrene (8 μg/ml). The infected cells were selected with puromycin (1 μg/ml) for at least 6 days before experiments.

### Transfection and reporter assays

The cells were transfected by standard calcium phosphate precipitation. To normalize for transfection efficiencies, pRL-TK (*Renilla* luciferase) reporter plasmid (0.02 μg) was added to each transfection. Empty control plasmid was added to ensure that each transfection receives the same amount of total plasmid DNA. Eighteen hours after transfection, cells were treated or left un-treated with the indicated stimuli before luciferase assays were performed using a dual-specific luciferase assay kit. Firefly luciferase activities were normalized based on *Renilla* luciferase activities.

### RNAi experiments

Double-strand oligonucleotides corresponding to the target sequences were cloned into the pSuper.Retro RNAi plasmid (Oligoengine). The following sequence of human *ZFYVE1* mRNA was targeted: GCGGACAGAGATTGTGCAT.

### qPCR

Total RNA was isolated from cells using the Trizol reagent. After reverse-transcription with oligo-dT primer using a First Strand cDNA Synthesis Kit, the samples were subjected to qPCR analysis to measure mRNA levels of the tested genes. Data shown are relative abundance of the indicated mRNA normalized to that of *GAPDH*. Sequences of qPCR primers were as following.

Human *GAPDH*: GACAAGCTTCCCGTTCTCAG, GAGTCAACGGATTTGGTGGT.

Human *ZFYVE1*: GTGCCCAAAACATCTGCTTCCAC, ACTGCCGACTACGATAGACCAC.

Human *IFNB1*: TGACTATGGTCCAGGCACAG,

TTGTTGAGAACCTCCTGGCT.

Human *ISG56*: TCATCAGGTCAAGGATAGTC,

CCACACTGTATTTGGTGTCTAGG.

Human *CXCL10*: GGTGAGAAGAGATGTCTGAATCC,

GTCCATCCTTGGAAGCACTGCA.

Murine *Gapdh*: ACGGCCGCATCTTCTTGTGCA, ACGGCCAAATCCGTTCACACC.

Murine *Zfyve1*: GAACGAGGACATACAATCCGCC, ACCGCTGAAGCGGTCACTTAGT.

Murine *Ifnb1*: TCCTGCTGTGCTTCTCCACCACA, AAGTCCGCCCTGTAGGTGAGGTT.

Murine *Isg56*: ACAGCAACCATGGGAGAGAATGCTG, ACGTAGGCCAGGAGGTTGTGCAT.

Murine *Il-6*: TCTGCAAGAGACTTCCATCCAGTTGC, AGCCTCCGACTTGTGAAGTGGT.

Murine *Cxcl10*: ATCATCCCTGCGAGCCTATCCT,

GACCTTTTTTGGCTAAACGCTTTC.

### *In vitro* pull-down assays

Poly(I:C)-HMW or 5'ppp-dsRNA was conjugated with biotin by UV (365-nm wavelength) cross-linking for 1 h. HEK293 cells transfected with the indicated plasmids were lysed in lysis buffer (20 mM Tris-HCl, pH 6.0, 150 mM NaCl, 1 mM EDTA, and 1% NP-40). The lysates were incubated with biotinylated-poly(I:C) at 37°C for 1 h and then incubated with streptavidin agarose at 37°C for additional 2 h. The beads were washed three times with lysis buffer and analyzed by immunoblots.

### Coimmunoprecipitation and immunoblotting analysis

Cells were lysed in 1 ml NP-40 lysis buffer (20 mM Tris-HCl, pH 7.4–7.5, 150 mM NaCl, 1 mM EDTA, 1% NP-40, 10 μg/ml aprotinin, 10 μg/ml leupeptin and 1 mM phenylmethylsulfonyl fluoride). The lysates were centrifuged at 12,000 rpm for 10 min at 4°C. For each immunoprecipitation, the supernatant was incubated with 0.5 μg of the indicated antibody and 35 μl of 50% slurry of GammaBind G Plus-Sepharose at 4°C for 2 h. The beads were then washed for three times with 1 ml lysis buffer containing 500 mM NaCl. The bound proteins were separated by SDS-PAGE and the associated proteins were analyzed by immunoblots.

### EMCV and VSV infection

Age- and sex-matched *Zfyve1*^*+/+*^ and *Zfyve1*^*-/-*^ mice were infected with EMCV and VSV through intraperitoneal (i.p.) route. The survival of the injected mice was monitored every 6 h. The blood was collected for ELISA measurement of cytokine levels after EMCV infection for 4, 6 and 8 h, or VSV infection for 6 h.

### CRISPR-Cas9 knockout

Genome engineering was performed using the CRISPR-Cas9 system [22, 23]. Double-stranded oligonucleotides corresponding to the target sequences were cloned into the lenti-CRISPR-V2 vector and co-transfected with psPAX2 and pMD2.G into 293 cells. Two days after transfection, the viruses were harvested and used to infect HEK293 cells. The infected cells were selected with puromycin (1 μg/ml) for at least 7 days to obtain ZFYVE1-KO cell pools. The following sequences were targeted for human ZFYVE1 mRNA: #1- TATACACTCGGTTGTCATAC; and #2-ACACTCGGTTGTCATACTGG.

### Preparation of recombinant proteins

To prepare recombinant GST-ZFYVE1, a cDNA encoding for ZFYVE1 was cloned into the pGEX-6p-1-GST plasmid. The plasmid was transformed into the *E*. *coli* BL21 strain. Expression of GST-ZFYVE1 was induced with 0.1 mM IPTG at 16°C for 24 h, which was purified with GST resins and eluted with elution buffer (PBS, 100 mM Tris-HCl pH 8.8, 40 mM reduced glutathione).

To prepare Flag-MDA5 protein, HEK293 cells (2 × 10^6^) were transfected with pRK-Flag-MDA5 plasmid (10 μg) for 24 h. The lysate was incubated with anti-Flag agarose beads for at 4°C for 2 h. The beads were washed three times with lysis buffer and Flag-MDA5 was then eluted with 3 × Flag peptide in 250 mM Tris-HCl, pH 8.0.

### RNA-binding protein immunoprecipitation (RIP)

HEK293 cells were transfected with the indicated HA-tagged plasmids for 20 h, then infected with EMCV or SeV for 1 h, washed with medium and cultured for additional 2 h. Cell lysates were immunoprecipitated with IgG or anti-HA (5 μg) and protein G beads (50 μl) at 4°C for 2 h. The immunoprecipitates were treated with diluted RNase I (1:25 in PBS) at 37°C for 5 min. The bead-bound immunoprecipitates were washed for three times with lysis buffer containing RNase inhibitors. The protein and RNA complexes were eluted with 200 μl TE buffer containing 10 mM DTT at 37°C for 30 min. The RNA was extracted using Trizol reagent before qPCR analysis for viral RNA levels.

### Viral plaque assays

Eight week-old mice were infected with EMCV or VSV for 4–6 days, the brains or lungs of mice were weighed and homogenized for 5 s in PBS. After homogenization, the brain or lung suspensions were centrifuged at 1,620 g for 30 min, and the supernatants were used for plaque assays. BHK21 or Vero cells were seeded in 24-well plates, and the cells were infected with serial dilutions of the brain or lung suspensions at 37°C for 2 h, overlaid with 2% methylcellulose and further incubated for 36–48 h. The overlay was then removed, and cells were fixed with 4% paraformaldehyde for 20 min and stained with 1% crystal violet for 20 min before plaque counting.

### Confocal microscopy

HEK293 cells were transfected with the indicated plasmids for 24 h, and then fixed with 4% paraformaldehyde for 10 min, and permeabilized with 0.1% Triton X-100 in PBS for 15 min. The cells were blocked with 1% BSA in PBS and stained with the indicated primary and secondary antibodies. The nuclei were stained with DAPI. The cells were observed with a Zeiss confocal microscope with a 60 × objective.

### Statistical analysis

Unpaired Student’s *t* test was used for statistical analysis with Microsoft Excel and GraphPad Prism Software. For the mouse survival study, Kaplan-Meier survival curves were generated and analyzed by Log-Rank test; *P* < 0.05 was considered significant.

## Supporting information

S1 Fig Binding of ZFYVE1, MDA5 or RIG-I to viral RNA. (A) ZFYVE1 and MDA5 bind to EMCV RNA. HEK293 cells were transfected with the indicated HA-tagged plasmids (5 μg each) for 20 h, then infected with EMCV for 1 h, washed with medium and cultured at 37°C for additional 2 h. Cell lysates were immunoprecipitated with IgG or anti-HA (5 μg) and protein G beads (50 μl) at 4°C for 2 h. The immunoprecipitates were treated with diluted RNase I (1:25 in PBS) at 37°C for 5 min. The bead-bound immunoprecipitates were washed for three times with lysis buffer containing RNase inhibitors. The protein and RNA complexes were eluted with 200 μl TE buffer containing 10 mM DTT at 37°C for 30 min. The protein-bound RNAs were extracted and analyzed by qPCR analysis with primers corresponding to the indicated regions of EMCV genome. (B) ZFYVE1, MDA5 and RIG-I bind to SeV RNA. HEK293 cells (2 × 10^6^) were transfected with the indicated plasmids (5 μg each). Twenty hours after transfection, cells were infected with SeV for 1 h. Cell lysates were collected for “footprint” experiments similarly as in (S1A). *P < 0.05, **P < 0.01 and ***P < 0.001 (unpaired t test). Data shown are mean ± SD, n = 3 (technical replicate, A and B), and representative of three biological replicates (A and B) with similar results.
(PDF)

S2 Fig A working model on the involvement of ZFYVE1 in MDA5-mediated signaling. ZFYVE1 is constitutively associated with MDA5 in un-infected condition. Upon viral infection, ZFYVE1 competes with MDA5 for viral RNA binding. The binding of ZFYVE1 to viral RNA causes its oligomerization and conformational changes, which reliefs its inhibition of MDA5.
(PDF)
