# Supplementary material for: ZFYVE1 negatively regulates MDA5- but not RIG-I-mediated innate antiviral response
Source: PLoS Pathog. 2020 Apr 6;16(4):e1008457. doi: 10.1371/journal.ppat.1008457 (PMC7162542; doi:10.1371/journal.ppat.1008457)
Supplement: S1 Fig — (A) ZFYVE1 and MDA5 bind to EMCV RNA. HEK293 cells were transfected with the indicated HA-tagged plasmids (5 μg each) for 20 h, then infected with EMCV for 1 h, washed with medium and cultured at 37°C for additional 2 h. Cell lysates were immunoprecipitated with IgG or anti-HA (5 μg) and protein G beads (50 μl) at 4°C for 2 h. The immunoprecipitates were treated with diluted RNase I (1:25 in PBS) at 37°C for 5 min. The bead-bound immunoprecipitates were washed for three times with lysis buffer containing RNase inhibitors. The protein and RNA complexes were eluted with 200 μl TE buffer containing 10 mM DTT at 37°C for 30 min. The protein-bound RNAs were extracted and analyzed by qPCR analysis with primers corresponding to the indicated regions of EMCV genome. (B) ZFYVE1, MDA5 and RIG-I bind to SeV RNA. HEK293 cells (2 × 10^6^) were transfected with the indicated plasmids (5 μg each). Twenty hours after transfection, cells were infected with SeV for 1 h. Cell lysates were collected for “footprint” experiments similarly as in (S1A). *P < 0.05, **P < 0.01 and ***P < 0.001 (unpaired t test). Data shown are mean ± SD, n = 3 (technical replicate, A and B), and representative of three biological replicates (A and B) with similar results. (PDF) [file ppat.1008457.s001.pdf]

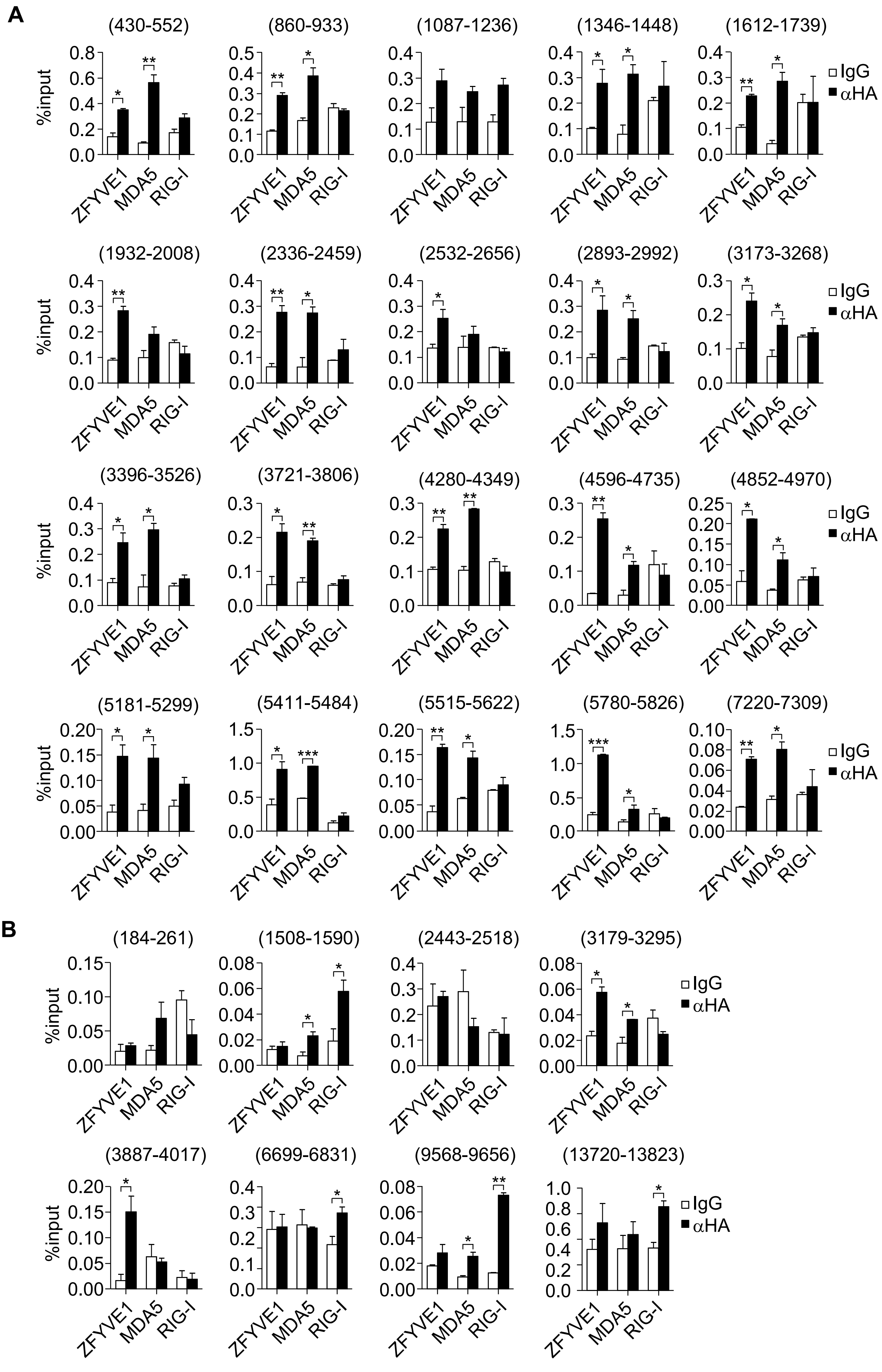
A
(430-552)
(860-933)
(1087-1236)
(1346-1448)
(1612-1739)
(1932-2008)
(2336-2459)
(2532-2656)
(2893-2992)
(3173-3268)
(3396-3526)
(3721-3806)
(4280-4349)
(4596-4735)
(4852-4970)
(5181-5299)
(5411-5484)
(5515-5622)
(5780-5826)
(7220-7309)
B
(184-261)
(1508-1590)
(2443-2518)
(3179-3295)
(3887-4017)
(6699-6831)
(9568-9656)
(13720-13823)
%input
ZFYVE1
MDA5
RIG-I
IgG
αHA

S1 Fig. Binding of ZFYVE1, MDA5 or RIG-I to viral RNA.
(A) ZFYVE1 and MDA5 bind to EMCV RNA. HEK293 cells were transfected with the indicated HA-tagged plasmids (5 μg each) for 20 h, then infected with EMCV for 1 h, washed with medium and cultured at 37°C for additional 2 h. Cell lysates were immunoprecipitated with IgG or anti-HA (5 μg) and protein G beads (50 μl) at 4°C for 2 h. The immunoprecipitates were treated with diluted RNase I (1:25 in PBS) at 37°C for 5 min. The bead-bound immunoprecipitates were washed for three times with lysis buffer containing RNase inhibitors. The protein and RNA complexes were eluted with 200 μl TE buffer containing 10 mM DTT at 37°C for 30 min. The protein-bound RNAs were extracted and analyzed by qPCR analysis with primers corresponding to the indicated regions of EMCV genome.
(B) ZFYVE1, MDA5 and RIG-I bind to SeV RNA. HEK293 cells ($2x10^6$) were transfected with the indicated plasmids (5 μg each). Twenty hours after transfection, cells were infected with SeV for 1 h. Cell lysates were collected for "footprint" experiments similarly as in (S1A).

*$P < 0.05$, **$P < 0.01$ and ***$P < 0.001$ (unpaired t test). Data shown are mean ± SD, n = 3 (technical replicate, A and B), and representative of three biological replicates (A and B) with similar results.
