# Supplementary material for: ZFYVE1 negatively regulates MDA5- but not RIG-I-mediated innate antiviral response
Source: PLoS Pathog. 2020 Apr 6;16(4):e1008457. doi: 10.1371/journal.ppat.1008457 (PMC7162542; doi:10.1371/journal.ppat.1008457)
Supplement: S2 Fig — ZFYVE1 is constitutively associated with MDA5 in un-infected condition. Upon viral infection, ZFYVE1 competes with MDA5 for viral RNA binding. The binding of ZFYVE1 to viral RNA causes its oligomerization and conformational changes, which reliefs its inhibition of MDA5. (PDF) [file ppat.1008457.s002.pdf]

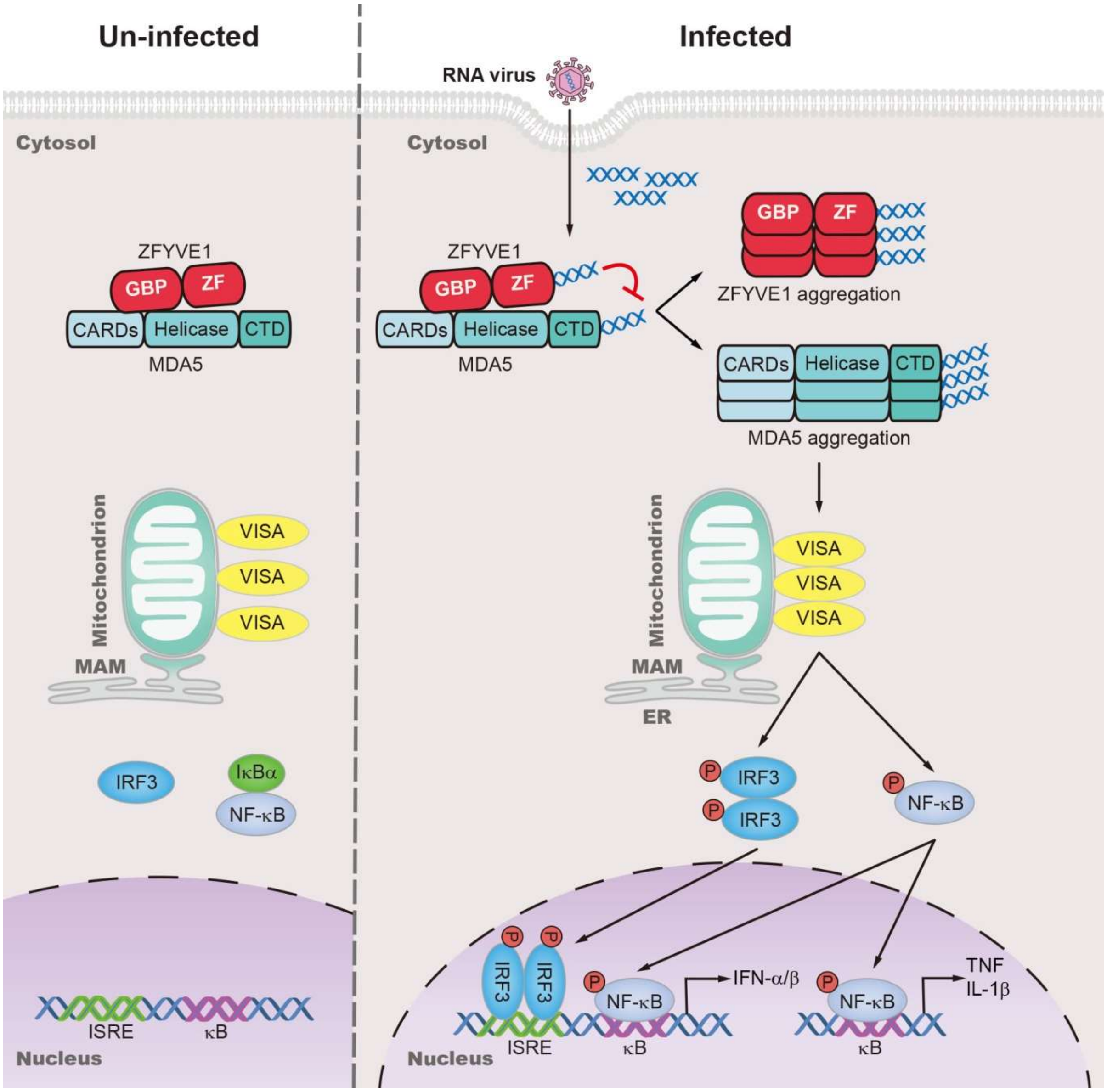

S2 Fig. A working model on the involvement of ZFYVE1 in MDA5-mediated signaling.

ZFYVE1 is constitutively associated with MDA5 in un-infected condition. Upon viral infection, ZFYVE1 competes with MDA5 for viral RNA binding. The binding of ZFYVE1 to viral RNA causes its oligomerization and conformational changes, which reliefs its inhibition of MDA5.
